# Free will belief predicts everyday punitiveness across 44 countries

**DOI:** 10.1111/bjso.70110

**Published:** 2026-07-19

**Authors:** Aaron Walton, Kelly Kirkland, Brock Bastian

**Affiliations:** ^1^ Melbourne School of Psychological Sciences University of Melbourne Parkville Victoria Australia; ^2^ School of Psychology The University of Queensland St. Lucia Queensland Australia

**Keywords:** agency, determinism, free will, moral responsibility, punishment, punitiveness, retribution

## Abstract

Belief in free will has been linked to more punitive and retributive responses to wrongdoing. While past research has focused on North American samples and criminal contexts, this study tested whether free will belief predicts punitiveness across diverse cultural contexts and focuses on more everyday moral transgressions. We surveyed 8917 participants from 44 countries using a validated measure of free will belief and a vignette‐based measure of punitiveness. Multilevel analysis revealed that individuals with stronger free will beliefs were more punitive towards everyday moral transgressions. These findings suggest that free will belief is a cross‐culturally robust predictor of punitiveness towards everyday moral transgressions. Implications are discussed for the potential benefits of mitigating free will belief in specific contexts.

Free will is the belief that individuals are ultimately free to choose their actions, that in a past moment of choice, they could have chosen differently in a way that was up to them (O'Connor & Franklin, 2022). The common antithesis to free will is determinism, which suggests that all events follow the laws of nature and arise from past events and states of the universe (Hoefer, 2024). Whether free will exists has long been debated among philosophers and psychologists. Regardless, however, of its metaphysical status, beliefs about free will are psychologically consequential, shaping how people interpret behaviour and how they respond to wrongdoing.

Beliefs about free will have important societal implications, and much research has suggested that reducing free will belief can produce negative consequences, including increased cheating (Vohs & Schooler, 2008), increased aggression and reduced helpfulness (Baumeister et al., 2009). Such outcomes have been used to argue that the public should be protected from the idea that free will is an illusion (Dennett, 2014; Vohs & Schooler, 2008). However, subsequent work has demonstrated that these behavioural effects seem to be fragile and context‐dependent (e.g. Buttrick et al., 2020; Nadelhoffer et al., 2020). This suggests (along with arguments that there are downsides to free will belief) that the psychological consequences of free will belief are unlikely to be consistently and uniformly beneficial, and more likely involve trade‐offs.

One potential trade‐off concerns punishment and retribution. There are potential downsides to a stronger belief in free will, one of which is a heightened focus on retribution and punitiveness rather than rehabilitation in response to transgressions (Carey & Paulhus, 2013; Gill & Cerce, 2017; Martin et al., 2017; Nettler, 1959; Shariff et al., 2014; Tygart, 1994). Strong free will belief may amplify the intuition that wrongdoers deserve suffering because they freely choose their actions, thereby strengthening retributive orientations. Understanding the link between free will belief and harsh retribution or punitiveness in response to transgressions has important implications for whether we should aim to protect such a belief or potentially mitigate it, at least within certain contexts where punitive responding carries high interpersonal or societal costs.

While research has focused on criminal transgressions, the question of whether to protect or mitigate the belief in free will also applies to much more common contexts in everyday life. Punitiveness and retribution are psychological phenomena expressed in everyday life: in how people respond to minor dishonesty, relationship violations, workplace slights and social norm breaches. Even when the wrongdoing is low‐level, punitive deservingness judgements can influence whether people seek repair, engage in rumination, retaliate or forgive. In this way, free will belief may shape moral judgement in a manner that affects interpersonal relationships and social cohesion, potentially influencing the escalation or de‐escalation of conflict at both interpersonal and intergroup levels (Fincham, 2000; Kelman, 2008; McCullough et al., 2003).

Here, we examine the relationship between free will belief and punitiveness, drawing on a large multinational sample and examining punitive responses within the context of everyday transgressions.

## Free will, moral responsibility and punitiveness

There is substantial evidence that free will belief is related to moral responsibility and outcomes that depend on it (e.g. Carey & Paulhus, 2013; Gill & Cerce, 2017; Krueger et al., 2014; Murray & Lombrozo, 2017; Nadelhoffer et al., 2020; Sarkissian et al., 2010). Free will belief is closely tied to the intuition that individuals could have acted otherwise and are therefore blameworthy when they violate moral norms. This intuition sits naturally within retributive conceptions of punishment, which focus on what wrongdoers deserve rather than on forward‐looking outcomes like deterrence or rehabilitation.

While reducing free will belief can lead to negative moral consequences, including increased cheating and aggression, and reduced helpfulness (Baumeister et al., 2009; Vohs & Schooler, 2008), emerging research has identified that reducing free will belief can also have intrapersonal and interpersonal benefits. Specifically, experimental work has shown that undermining free will belief can increase forgiveness of self and others and reduce hatred following wrongdoing (Walton et al., 2026). These findings suggest that free will belief may be psychologically double‐edged: potentially supporting certain forms of norm compliance or self‐control in some contexts while also amplifying moral blame, harsh moral judgements and emotions, and punitive responding in other contexts.

Consistent with this, it has been demonstrated that higher free will belief is related to harsher punitive responses to wrongdoing (e.g. Carey & Paulhus, 2013; Gill & Cerce, 2017; Martin et al., 2017; Nettler, 1959; Shariff et al., 2014; Tygart, 1994), and eagerness for retribution rather than rehabilitation in response to criminal behaviour (Carey & Paulhus, 2013; Nettler, 1959). Providing additional support for this relationship, experimental studies have shown that reducing free will belief leads to reductions in endorsement of spiteful and retributive punishment and harsh blame (Gill & Cerce, 2017; Shariff et al., 2014). Notably, these effects appear selective. For example, undermining free will belief reduces endorsement of punishment aimed at inflicting suffering for its own sake, but does not necessarily reduce support for instrumental punishment aimed at deterrence or harm reduction (Gill & Cerce, 2017; Shariff et al., 2014). Similarly, experimental evidence shows that undermining belief in free will increases forgiveness of self and others and reduces hatred, without necessarily reducing judgements of personal accountability regarding oneself or others (Walton et al., 2026). Together, these findings are important because they directly address the concern that weakening belief in free will might broadly undermine accountability or pragmatic justice.

The relationship between free will belief and punitiveness also extends to endorsement of the death penalty, with several studies showing free will belief relates to stronger endorsement of the death penalty (Martin et al., 2017; Nettler, 1959; Tygart, 1994). This is consistent with the idea that capital punishment represents a particularly strong expression of retributive reasoning: an extreme ‘just deserts’ response for the worst transgressions.

The broad pattern that free will belief generally predicts higher endorsement of punishment, particularly retributive punishment, is consistent with theoretical formulations. Carlsmith and Darley (2008) argue that the goal of retributive justice is to give people their ‘just deserts’, and that ‘any circumstance that mitigates responsibility for the action would also mitigate the moral severity of the offense’ (Carlsmith & Darley, 2008, p. 210). High levels of free will imply that a person can do otherwise, and therefore is blameworthy for their actions, providing a justification for retributive punishment, that is inflicting suffering because it is deserved, not because it will produce forward‐looking benefits like deterrence.

## The current study

While existing evidence suggests a relationship between free will belief and retributive or punitive responses to transgressions, there is no known evidence showing this relationship exists across a diverse range of national contexts and in response to everyday forms of transgression. Our aim was to provide a robust test of this association, drawing on a large multinational data set. Previous research in this area has largely focused on criminal transgressions within North American samples, with only two exceptions. One study by Gill and Cerce (2017) explored a non‐criminal context, investigating how information about an office bully's difficult past influenced retributive attitudes towards him. Their findings showed that reducing perceptions of the bully's ‘control of self‐formation’—a proposed facet of free will belief—lowered punitiveness. However, this study focused on a narrow aspect of free will belief and relied on participants from Amazon's Mechanical Turk, a platform heavily weighted towards US respondents. Another study by Martin et al. (2017) examined a more culturally diverse sample, but used a limited two‐item proxy measure to assess free will belief and focused exclusively on endorsement of the death penalty, hence focusing on an extreme criminal context. The current study builds on and extends this prior work by examining, for the first time, whether general belief in free will predicts punitive responses to everyday minor moral transgressions. We do so using a large, diverse sample spanning 44 countries and employing a validated measure of free will belief. We predicted that higher belief in free will would be related to greater everyday punitiveness at the individual level.

Furthermore, to replicate and extend previous research, we examined relationships between free will belief and a country‐level indicator of punitiveness: endorsement of the death penalty. This link has been demonstrated multiple times at the individual level (Nettler, 1959; Tygart, 1994), including once multinationally (Martin et al., 2017). We sought to examine whether country‐level averages of free will belief would predict country‐level averages of death penalty endorsement in the World Values Survey Dataset. Given that this test is done at a distinct level of analysis (country‐level averages rather than individual responses), we consider it an exploratory conceptual extension, despite being pre‐registered. Further, we express caution in interpretation, given the small sample size.

## METHOD

This study was pre‐registered with AsPredicted. We preregistered the primary hypothesis that free will belief would predict everyday punitiveness (at the individual level) as well as the more exploratory test of the relationship between (country‐mean) free will belief and (country‐mean) endorsement of the death penalty. This analysis was motivated by prior individual‐level multinational findings reported by Martin et al. (2017). However, because this analysis involved aggregation to the country level and relied on a limited number of countries with available data, it represents an exploratory conceptual extension of prior work, despite being preregistered.^1^

### Participants

A total of 8917 participants were recruited across 44 countries (see Supporting Information for a list of countries and country sample sizes) using Dynata, a global data collection company. Participants were required to be at least 18 years old and fluent in the primary language of their country. The final sample included individuals from a wide range of cultural, economic and political contexts, providing a robust basis for cross‐national comparisons. This dataset has not been previously used to examine everyday punitiveness. Although subsets of the broader dataset have been analysed in separate unpublished manuscripts by other researchers addressing distinct research questions, those analyses are clearly delineated and do not overlap with the outcomes reported here.

### Materials & procedure

Participants were invited to complete an online survey hosted on Dynata's data collection platform. The survey comprised multiple measures for this and several other unrelated studies. For this study, materials included the Free Will and Determinism subscales of the Free Will Inventory and a measure of punitive attitudes. Participants provided informed consent before beginning the survey and were compensated according to Dynata's standard rates for their country. Survey materials were translated from English into the dominant language of each non‐English‐speaking country by a paid external translation service through Dynata (using human translators). We then, as a team of researchers, checked these outputs with back‐translations generated using OpenAI's GPT‐4 model and manually checked for errors. Furthermore, the translations for French, Chinese, Spanish and Korean were additionally verified by fluent or native speakers.

### Free will belief

The five‐item Free Will subscale of the Free Will Inventory (Nadelhoffer et al., 2014) was used to assess participants' belief in free will. Items include ‘People have complete free will’. Participants rated each item on a 5‐point Likert scale ranging from 1 (strongly disagree) to 5 (strongly agree). The Free Will subscale has demonstrated reliability and validity and, in the current study, demonstrated sound internal consistency (*α* = .78).

### Everyday punitiveness

To measure everyday punitiveness, participants were presented with a series of ten short vignettes detailing relatively everyday moral transgressions, for example ‘*I was on vacation and rented a car. I paid for the parking fee and the guy gave me the wrong change back. I knew he gave me too much, but I kept it anyway*’. and ‘*As I was backing out of a parking lot, I bumped a parked car and left a minor dent. I didn't even feel the impact when I hit the car, but it left a little bit of damage. I drove away without leaving a message or trying to contact the person*’. At the end of each vignette, they were asked three questions, including the question used for our measure: ‘How deserving is this person of punishment?’. These vignettes were sourced from Kruepke et al. (2018). Participants answered on a 7‐point Likert scale from, ‘Not at all’ to ‘Very much’ (the full three items measure moral judgements, which were not the focus of our work). Everyday punitiveness was operationalized as the mean of the 10 vignette items (*α* = .83).

### Endorsement of the death penalty

Data on countries' endorsement of the death penalty were obtained from the latest wave of the World Values Survey (WVS, 2022), which included a single item: ‘*Please tell me for each of the following statements whether you think it can always be justified, never be justified, or something in between, using this card. Death penalty*’. Responses were recorded on a 10‐point scale (1 = Never justifiable, 10 = Always justifiable, with an option to select ‘Don't know’). This item was included to examine the relationship between free will belief and support for capital punishment.

## APPROACH TO ANALYSES

Analyses were conducted in R 4.4.0 using the *lme4* package (Bates et al., 2015).

### Punitive attitudes

Punitive attitudes were analysed using multilevel linear modelling because both free will belief and punitive attitudes were measured at the individual level, with participants nested within countries. Punitive attitudes served as the dependent variable, and free will belief served as the primary independent variable. The country was included as a random intercept to account for clustering of individuals within countries. Free will belief was decomposed into within‐ and between‐country components, with individual deviations from the country mean capturing within‐country effects, and country‐level means capturing between‐country effects. Both components were entered simultaneously as predictors. All continuous individual‐level predictors were standardized prior to analysis to facilitate comparability of effect sizes. Age, gender, religiosity and political orientation were included as individual‐level covariates.

### Death penalty endorsement

Because endorsement of the death penalty was available only at the country level, multilevel models could not be estimated for this outcome. Instead, an ordinary least squares regression analysis was conducted. In this model, the mean country‐level free will belief served as the independent variable and country‐level endorsement of the death penalty was the dependent variable. Country‐level predictors were analysed on their original scales and were not standardized. Standard model assumptions were evaluated for this regression. Given the limited number of countries (*N* = 44), no additional country‐level covariates were included in the model to avoid overfitting and preserve statistical power.

## RESULTS

**FIGURE 1:**
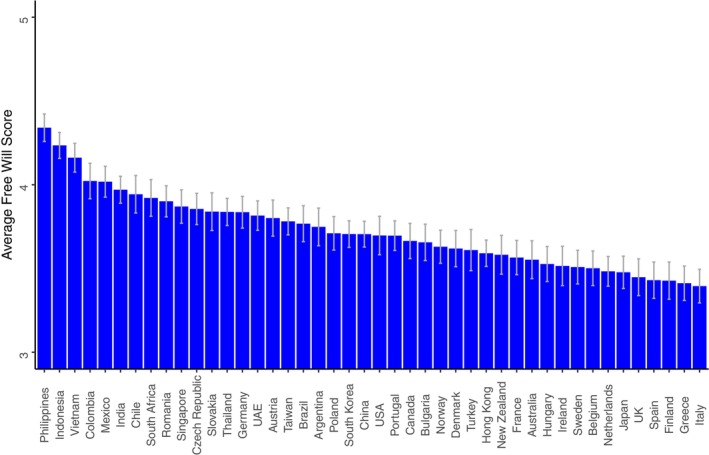
Average free will belief score across locations. Higher values indicate greater belief in free will. Grey bars represent confidence intervals.

The descriptive statistics for all variables can be found in Table 1 below. Based on the intraclass correlation, approximately 8.6% of the variance in free will belief can be explained by differences between countries (see Figure 1).

**TABLE 1:** Descriptive statistics of all variables.

Variable	Range	*M*	SD
Free will belief	1–5	3.72	0.75
Punitiveness	1–7	4.33	1.12
Death penalty endorsement (*N* = 38)	2.31–6.96	3.85	1.01

*Note*: *N* = 8917 individuals across 44 countries. Free will belief and punitiveness are individual‐level variables. Death penalty endorsement is a country‐level variable.

### Punitiveness

**FIGURE 2:**
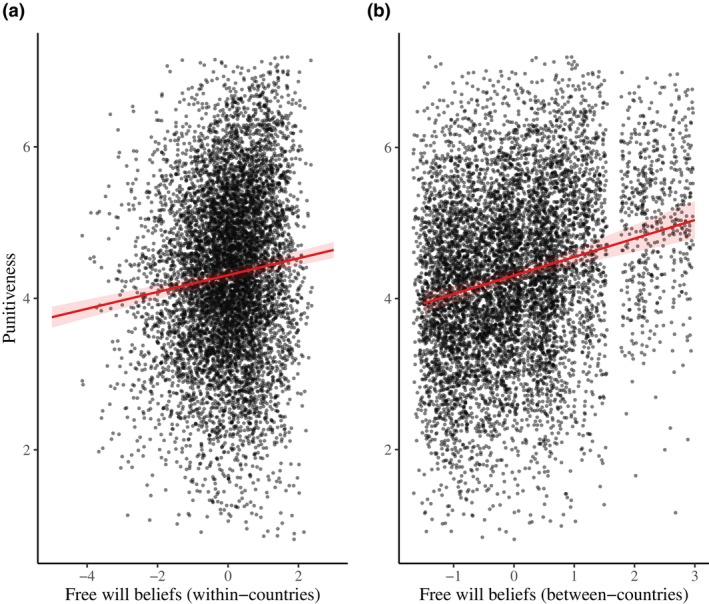
The relationship between free will beliefs and punitiveness within countries (panel A) and between‐countries (panel B). Variables have been scaled and centred. Each point has been jittered for ease of interpretation and the light red shaded area surrounding the trend line represents confidence intervals.

**FIGURE 3:**
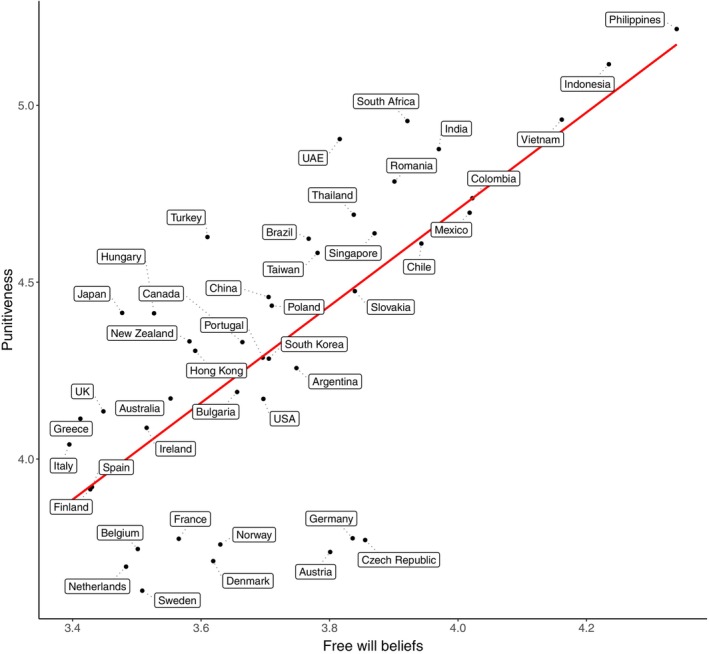
The relationship between the average free will beliefs and punitiveness by country.

A linear mixed‐effects model was conducted to examine the association between free will belief (within‐ and between‐country components) and punitiveness, controlling for age, gender, religiosity and political orientation (see Table 2). As shown in Figures 2 and 3, stronger belief in free will was associated with harsher punishment judgements at both the within‐ and between‐country levels. Older age, greater religiosity and stronger social conservatism were also associated with increased punitiveness. No significant effects were observed for gender or economic conservatism.

**TABLE 2:** Linear mixed model examining the effect of free will belief on punitiveness.

Predictors	*b*	95% CI	*p*
Intercept	4.31	4.22–4.39	< .001***
Free will belief (within countries)	0.11	0.09–0.13	< .001***
Free will belief (between‐countries)	0.24	0.16–0.33	< .001***
Gender (female)	0.04	−0.00 – 0.09	.052
Age	0.07	0.04–0.09	< .001***
Importance of religion	0.18	0.15–0.20	< .001***
Economic conservativism	0.00	−0.03 to 0.03	.936
Social conservativism	0.05	0.02–0.09	.002**
**Random effects**			
Residual	1.02		
Country (intercept)	0.07		
ICC	.06		
Observations	8150		
Marginal *R* ^2^/conditional *R* ^2^	.122/.175		

*Note*: Gender was coded as male (1) and female (2). Marginal *R* ^2^ refers to fixed effects only, and Conditional *R* ^2^ refers to the entire model. Predictors were standardized prior to analysis; coefficients represent the expected change in punitiveness associated with a one standard deviation increase in the predictor. *p < .05, **p < .01, ***p < .001.

### Death penalty endorsement

A linear regression revealed no significant effect of mean free will belief on citizen endorsement of the death penalty, *b* = 0.65, SE = 0.74, *p* = .385. Inspection of residual plots and formal diagnostic tests indicated no violations of linearity, normality, homoscedasticity or independence assumptions.

### Measurement invariance

We tested measurement invariance for our two key constructs (free will belief and everyday punitiveness) across all countries to ensure comparability. Both scales demonstrated metric invariance, indicating that the relationships between items and their underlying constructs were consistent across groups. However, scalar invariance was not supported, meaning that latent means may not be directly comparable across countries. While metric invariance is sufficient for the individual‐level correlational and regression analyses we conduct, some caution is warranted when interpreting country‐level relationships involving aggregated scores (free will belief), as these may reflect measurement differences across countries. Full measurement invariance results are provided in the Supporting Information.

## DISCUSSION

In the current study, we examined the relationship between free will belief and everyday punitiveness across a diverse, multinational sample. Surveying 8917 participants across 44 countries, we found that individual differences in free will belief predict more punitive responses to everyday moral transgressions. This relationship remained statistically significant after controlling for age, gender, religiosity and political ideology. These findings provide the first large‐scale cross‐cultural evidence that stronger belief in free will is associated with greater punitiveness in response to commonplace moral violations.

At the societal level, we failed to extend prior research (which demonstrated that individual‐level free will belief predicts support for the death penalty; Martin et al., 2017), finding no significant relationship between country‐level mean free will belief and national attitudes towards the death penalty (using data from the World Values Survey). Given the limited number of countries with available data in both the World Values Survey and our current dataset, and the resulting lack of statistical power, this null finding should be interpreted cautiously. Importantly, the absence of a country‐level association does not undermine the individual‐level results, as psychological associations observed at the individual level do not necessarily scale to higher‐order aggregates. Overall, the primary contribution of the present study lies in its well‐powered test of individual‐level associations between belief in free will and everyday punitiveness across a wide range of national contexts.

### Free will belief, moral responsibility and retributive judgement

Our findings align with a substantial body of previous research showing that stronger belief in free will is associated with greater punitive and retributive responses to criminal wrongdoing (Carey & Paulhus, 2013; Confer & Chopik, 2019; Gill & Cerce, 2017; Martin et al., 2017; Nettler, 1959; Shariff et al., 2014; Tygart, 1994). This effect appears to be driven by heightened attributions of moral responsibility (Carey & Paulhus, 2013; Roskies & Nichols, 2008; Sarkissian et al., 2010). When individuals are perceived as having freely chosen their actions, they are more likely to be judged blameworthy and deserving of punishment (Gill & Cerce, 2017; Roskies & Nichols, 2008; Sarkissian et al., 2010). This sense of deservingness is a defining aspect of retribution.

Retributive justice is grounded in the principle of just deserts: the notion that wrongdoers deserve to suffer because they have committed a moral transgression, independent of any forward‐looking benefits such as deterrence or rehabilitation (Carlsmith & Darley, 2008; Walen, 2024). Belief in free will provides a psychological foundation for this principle by reinforcing the intuition that individuals could have acted otherwise and are therefore fully responsible for their actions. From this perspective, the observed association between free will belief and punitiveness reflects a deeper alignment between metaphysical intuitions about agency and moral judgements about blame and punishment.

Crucially, prior experimental work suggests that this relationship is selective rather than indiscriminate. Reducing belief in free will decreases endorsement of punishment aimed at inflicting suffering for its own sake, but does not necessarily reduce support for punishment aimed at instrumental or consequentialist goals such as deterrence or harm prevention, either among lay persons (Gill & Cerce, 2017; Shariff et al., 2014), nor among professional criminal judges Genschow et al. (2021). Converging experimental evidence further indicates that undermining belief in free will increases forgiveness of self and others and reduces hatred, without necessarily diminishing judgements of personal accountability for one's own or others' actions (Walton et al., 2026).

The present findings extend this line of work by showing that individual differences in free will belief predict punitive deservingness judgements in response to everyday moral transgressions across a large and diverse multinational sample.

### Extending the free will–punitiveness link beyond criminal contexts

A central contribution of the present study is the demonstration that free will belief predicts punitive judgements in response to relatively minor everyday moral transgressions. Whereas much prior research has focused on severe criminal behaviour or abstract policy attitudes, everyday moral violations are psychologically consequential because they occur frequently and often within ongoing social relationships. How individuals interpret and respond to such violations shapes patterns of conflict, reconciliation and social cohesion.

The finding that free will belief predicts harsher judgements of deservingness in these contexts suggests that the cognitive and moral processes linking free will belief to punishment are not confined to criminal contexts. Instead, they appear to operate broadly across moral life, influencing how people evaluate wrongdoing in mundane situations such as dishonesty, norm violations or failures of consideration. This extension is theoretically important, as it indicates that free will belief may shape moral judgement in ways that accumulate over time through repeated interpersonal interactions.

These findings also complement prior work by Gill and Cerce (2017), who demonstrated that reducing perceived control over self‐formation decreases punitiveness in a non‐criminal context. The present study extends this work by examining a wider range of everyday transgressions, using a validated measure of general free will belief and demonstrating the robustness of the association across a large number of national contexts.

### Implications for interpersonal relationships and forgiveness

Beyond punishment attitudes, the present findings have important implications for interpersonal relationships. Judgements concerning whether a transgressor deserves punishment are closely linked to reduced forgiveness, heightened anger and difficulties in relationship repair (Fincham, 2000; McCullough, 2001). The more that wrongdoing is attributed to free will, the more likely individuals are to engage in harsh blame (Gill & Cerce, 2017; Shariff et al., 2014), which in turn predicts lower willingness to forgive and poorer relational outcomes following transgressions (Finkel et al., 2002).

Common moral transgressions (such as those examined in the present study) are consequential because they often occur within close relationships and accumulate over time. Even relatively minor violations can escalate into cycles of rumination, resentment and retaliation when wrongdoing is interpreted through a desert‐based lens that emphasizes blameworthiness and moral entitlement to punishment (Carlsmith, 2006; Carlsmith & Darley, 2008; McCullough et al., 2007). From this perspective, a stronger belief in free will may bias individuals towards punitive moral appraisals that undermine reconciliation and repair, even when the relational costs of punishment outweigh its benefits.

Importantly, experimental evidence converges with this interpretation. In prior experimental work, undermining belief in free will increased forgiveness (of both self and others) and reduced hate following real‐life interpersonal harms (Walton et al., 2026). Although these harms were not limited to trivial norm violations, they overwhelmingly involved relatively minor interpersonal transgressions rather than criminal wrongdoing, suggesting that the psychological processes linking free will belief to blame and forgiveness operate within ordinary social life.

The present findings complement and extend this experimental work by demonstrating that, at the individual level, stronger belief in free will is associated with greater punitive deservingness judgements in response to standardized everyday moral transgressions across diverse national contexts. Taken together, this body of evidence suggests a coherent pattern: stronger endorsement of free will predicts harsher moral evaluation and punishment attitudes, whereas experimentally undermining free will belief promotes forgiveness and reduces hostile responses following interpersonal wrongdoing.

Future experimental work could productively integrate these approaches by directly manipulating belief in free will and examining its effects specifically on punitive deservingness judgements in response to everyday moral transgressions. Such work would allow for causal tests of the mechanisms suggested by the present correlational findings and would further clarify how free will belief shapes moral responses to common interpersonal harms.

Our findings failed to extend those of Martin et al. (2017) to the country level, finding that free will belief is not significantly associated with endorsement of the death penalty. Several factors likely contributed to this result. First, the effective sample size for country‐level analyses was limited to the number of countries with available data, substantially reducing statistical power. Future research could examine this with a larger country sample. Second, a lack of measurement invariance across countries limits the interpretability of aggregated latent means. Third, country‐level punishment policies and attitudes are shaped by a wide range of historical, political and institutional factors that may attenuate or obscure psychological relationships observed at the individual level.

Importantly, null findings at the country level are compatible with robust individual‐level associations. Psychological processes that influence individual moral judgement do not necessarily translate directly into national policies or cultural norms. Accordingly, the primary contribution of the present study lies in its well‐powered test of individual‐level relationships between free will belief and everyday punitiveness across diverse cultural contexts.

Our findings also extend on this past work by moving beyond the context of criminal behaviour and showing that free will belief is also associated with punitive responses to minor everyday transgressions. Whereas Gill and Cerce (2017) have also shown the free will–punitiveness link in non‐criminal contexts, our work extends on this by examining a larger range of everyday transgressions and demonstrating this relationship across 44 national contexts.

## LIMITATIONS AND FUTURE DIRECTIONS

Several limitations should be noted. First, while our study used a large and diverse sample, it remains correlational in nature. Further, belief in free will and punitive attitudes were measured at the same time point. This means that while we have demonstrated robust associations between free will belief and punitive attitudes, we cannot infer causal relationships with our data. Future experimental and longitudinal research could clarify the directionality and causal mechanisms underlying these associations. It should, however, be noted that our results are consistent with previous experimental work showing that undermining free will belief reduces endorsement of spiteful, retributive punishment (Gill & Cerce, 2017; Shariff et al., 2014).

A further limitation is that while we employed a validated measure of free will belief, this belief is complex and difficult to measure. Future studies should seek to more clearly examine how different varieties of free will belief (specifically, belief in libertarian vs. compatibilist free will; see O'Connor & Franklin, 2022) may differentially influence punitive attitudes.

Moreover, our findings are limited by the use of personal reports. Future research should explore how free will belief relates to behavioural measures of punitiveness. Finally, while our measure of punitiveness focused on deservingness, the defining aspect of retribution, it does not expressly distinguish between retribution and consequentialist punishment. As stated, previous research does make this distinction clearly, and our results are consistent with their findings, but future research, both in a multinational context and on everyday moral transgressions, should seek to more clearly distinguish these motivations for punishment.

## CONCLUSION

The present study provides evidence that belief in free will is positively associated with harsher punitive attitudes towards everyday moral transgressions across diverse national contexts. These findings indicate that free will belief covaries not only with abstract views about punishment and justice but also with everyday moral evaluations that are likely to influence how individuals respond to wrongdoing in ordinary social life.

Although the present data are correlational and therefore do not permit causal conclusions, the observed associations are consistent with prior experimental research showing that reducing belief in free will can increase forgiveness and reduce retributive hostility without necessarily diminishing judgements of personal accountability. Taken together, the present findings are consistent with emerging arguments and empirical work suggesting that, alongside the potential downsides of lower free will belief reported in prior literature (e.g. increased cheating and aggression; Vohs & Schooler, 2008; Baumeister et al., 2009; though see Buttrick et al., 2020), reduced belief in free will may also be associated with certain adaptive outcomes, particularly in interpersonal contexts where moral evaluation and responses to everyday wrongdoing play a central role.

As the idea that free will may be an illusion continues to gain mainstream exposure, understanding how free will beliefs relate to moral judgement has important implications not only for justice systems but also for how people conceive of agency, accountability, interpersonal relationships and the moral foundations of social life.

## AUTHOR CONTRIBUTIONS

**Aaron Walton:** Conceptualization; methodology; data curation; investigation; formal analysis; project administration; writing – original draft; writing – review and editing. **Kelly Kirkland:** Data curation; formal analysis; writing – review and editing. **Brock Bastian:** Conceptualization; methodology; writing – review and editing; supervision; investigation; resources.

## CONFLICT OF INTEREST STATEMENT

The authors declare no competing interests.

## OPEN PRACTICES

Pre‐registration: Our studies were pre‐registered before data collection began, anonymous preregistration is available here: https://aspredicted.org/kpbz‐phtp.pdf.

## Supporting information


**Table S1:** Linear mixed model examining the effect of free will belief on moralizing.
**Figure S1:** The relationship between free will beliefs and moralizing within‐countries (panel A) and between‐countries (panel B). Variables have been scaled and centred. Each point has been jittered for ease of interpretation and the light red shaded area surrounding the trend line represents confidence intervals.
**Table S2:** Linear mixed model examining the effect of free will belief on punitiveness.

## Data Availability

The data that support the findings of this study are openly available in The Open Science framework at https://osf.io/9z4mr/?view_only=908d25917e884d61b6d93ca050b5dda9.

## Footnotes

^1^
We also pre‐registered that free will belief would predict moralizing. This result was also significant as hypothesized (See Supporting Information). We note that the preregistration specified Pearson correlations as the primary analytic approach. After preregistration, but prior to data analysis, we recognized that this approach did not adequately account for the hierarchical structure of the data (individuals nested within countries). Accordingly, the analytic strategy was revised to use multilevel modelling, which more appropriately reflects the structure of the data and provides more accurate parameter estimates.
