# Supplementary material for: Free will belief predicts everyday punitiveness across 44 countries
Source: Br J Soc Psychol. 2026 Jul 19;65(3):e70110. doi: 10.1111/bjso.70110 (PMC13382202; doi:10.1111/bjso.70110)
Supplement: Supplementary file 1 — Table S1: Linear mixed model examining the effect of free will belief on moralizing. Figure S1: The relationship between free will beliefs and moralizing within‐countries (panel A) and between‐countries (panel B). Variables have been scaled and centred. Each point has been jittered for ease of interpretation and the light red shaded area surrounding the trend line represents confidence intervals. Table S2: Linear mixed model examining the effect of free will belief on punitiveness. [file BJSO-65-0-s001.docx]

Supplementary Materials

**Free Will Measure**

The Free Will subscale of the Free Will Inventory

Sourced from:

Nadelhoffer, T., Shepard, J., Nahmias, E., Sripada, C., & Ross, L. T. (2014). The free will inventory: Measuring beliefs about agency and responsibility. *Consciousness and cognition*, *25*, 27-41.

Indicate the degree to which you agree with the following statements.

- People always have the ability to do otherwise.
- People always have free will.
- How people’s lives unfold is completely up to them.
- People ultimately have complete control over their decisions and their actions.
- People have free will even when their choices are completely limited by external circumstances.

Each item was answered on the following 5-point Likert scale:

| Strongly disagree (1) | Disagree  (2) | Neither disagree nor agree (3) | Agree  (4) | Strongly agree (5) |
| --- | --- | --- | --- | --- |
|  |  |  |  |  |

Scores for each item were summed to give a total score for free will belief.

**Moralising Measure**

Moral Vignettes

Sourced from:

Kruepke, M., Molloy, E. K., Bresin, K., Barbey, A. K., & Verona, E. (2018). A brief assessment tool for investigating facets of moral judgment from realistic vignettes. *Behavior Research Methods*, *50*(3), 922–936. https://doi.org/10.3758/s13428-017-0917-3

Participants were presented with 10 short vignettes describing morally relevant behaviours. After reading each vignette, they answered three questions assessing their moral evaluations of the behaviour and the person involved: (1) “How morally wrong is it?”, (2) “How deserving is this person of punishment?”, and (3) “Does this behaviour reveal a lot about this person’s character?” Responses were recorded on 7-point Likert scales ranging from 1 (*Not at all*) to 7 (*Very much*), with 4 (*Somewhat*) as the midpoint (α = .91). Moralising (overall moral judgment) was operationalised as the mean of all 30 moral-evaluation responses (3 items × 10 vignettes). Higher scores indicate stronger tendency to moralise.

Before completing the task, participants were given the following instruction: “The following scenario is a real-life account written by a real person. Please read the scenario and answer how much you agree with the following questions.” The ten vignettes were as follows:

1. I used to work as a legal assistant. I lied to clients all of the time, the attorney I worked for lied all the time too. We just lie to our clients so things will work out the best for us.
2. I was in a relationship with this woman named Beth for a while. Then one night I cheated on Beth with my ex-girlfriend. The next morning my ex came by to see why I didn't stay the night at her place, and she saw me with Beth.
3. I took my sister's ring that was given to her by our father's aunt. I had never met our father’s aunt, but my sister spent some time with her. I took the ring because I didn't think that my sister deserved it.
4. When my mother was dying, she wanted to go out to lunch with me and a bunch of her friends. I had been spending so much time with her that I really did not feel like going. So, I told her that I needed some space for myself.
5. I said some bad things about this guy that I work with. He told me some secrets that I promised not to share, but I did anyway. He didn’t find out, but I was afraid if he did, he might do something crazy.
6. As I was backing out of a parking lot, I bumped a parked car and left a minor dent. I didn’t even feel the impact when I hit the car, but it left a little bit of damage. I drove away without leaving a message or trying to contact the person.
7. I was on vacation and rented a car. I paid for the parking fee and the guy gave me the wrong change back. I knew he gave me too much, but I kept it anyway.
8. Back in high school I kind of had an agreement with the guy sitting next to me. We would show each other our papers whenever we were taking a test. Both of us were pretty good students, we just would make sure we shared test answers if we needed to.
9. My friends and I were going to the movies, and I was taking this guy with me in my car. He was being a jerk about my driving, so I decided to go really fast and drive recklessly. It got to the point where he told me to stop the car so he could get out.
10. My sister is five years older than I am. When we were little, she would always tease me about things. So, one day when she was teasing me about something, I threw a pencil at her.

**Results for Full Moralising Measure**

A linear mixed-effects model was conducted to examine the association between free will belief (within- and between-country components) and moralising, controlling for age, gender, religiosity, and political orientation (see Table S1). As shown in Figure S1, stronger belief in free will was associated with a greater tendency to moralise at both the within- and between-country levels. Older age, greater religiosity and stronger social conservatism were also associated with increased punitiveness. Moreover, females (*M* = 4.91, *SD* = 0.88) tended to moralize at a slightly higher rate relative to males (*M* = 4.83, *SD* = 0.89). No significant effects were observed for economic conservatism.

**Table S1**

*Linear Mixed Model Examining the Effect of Free Will Belief on Moralising*

| ***Predictors*** | ***b*** | | ***95% CI*** | | ***p*** |
| --- | --- | --- | --- | --- | --- |
| (Intercept) | 4.82 | 4.75 – 4.90 | | | <.001 |
| Free will belief (within-countries) | 0.17 | 0.10 – 0.24 | | | <.001 |
| Free will belief (between-countries) | 0.13 | 0.11 – 0.14 | | | <.001 |
| Gender (female) | 0.10 | 0.06 – 0.13 | | | <.001 |
| Age | 0.06 | 0.04 – 0.08 | | | <.001 |
| Importance of religion | 0.14 | 0.12 – 0.16 | | | <.001 |
| Economic conservativism | 0.00 | -0.02 – 0.03 | | | .821 |
| Social conservativism | 0.04 | 0.01 – 0.06 | | | .007 |
| ***Random Effects*** |  |  | | |  |
| Residual | 0.63 |  | | |  |
| Country (intercept) | 0.05 |  | | |  |
| ICC | .07 |  | | |  |
| Observations | 7954 | | |  |  |
| Marginal R^2^ / Conditional R^2^ | .123 / .188 | | |  |  |

*Note:* Gender was coded as male (1) and female (2). Marginal R^2^ refers to fixed effects only and Conditional R^2^ refers to the entire model. Predictors were standardized prior to analysis; coefficients represent the expected change in punitiveness associated with a one standard deviation increase in the predictor.

* *p* < .05 ** *p* < .01 *** *p* < .001

*Figure S1.* The relationship between free will beliefs and moralising within-countries (panel A) and between-countries (panel B). Variables have been scaled and centered. Each point has been jittered for ease of interpretation and the light red shaded area surrounding the trend line represents confidence intervals.

**Measurement Invariance**

We conducted measurement invariance testing on our two key psychometric constructs (free will belief and punitiveness) to assess whether these measures function equivalently across countries. Multi-group confirmatory factor analysis (CFA) was conducted using the MLR estimator, following the standard three-step sequence: configural, metric, and scalar models.

**Free Will Belief** A one-factor CFA model was specified for the free will belief scale and tested across all 44 countries. The configural model demonstrated good fit (CFI = .970, RMSEA = .084, SRMR = .034), indicating that the basic factor structure was consistent across groups. The metric invariance model also showed acceptable fit (CFI = .960, RMSEA = .073, SRMR = .062), and the changes relative to the configural model (ΔCFI = −.0097; ΔRMSEA = −.0116; ΔSRMR = +.0284) fell within recommended thresholds (Chen, 2007), supporting equivalence of item-factor loadings across countries. In contrast, scalar invariance was not supported. The scalar model showed a substantial drop in fit (CFI = .834, RMSEA = .124, SRMR = .103), with all Δ values exceeding conventional cut-offs (ΔCFI = −.127; ΔRMSEA = +.052; ΔSRMR = +.0408), suggesting that item intercepts varied across countries.

These findings indicate that the free will belief scale demonstrates metric but not scalar invariance. This supports the validity of cross-national comparisons of regressions or correlations involving free will belief but does not support direct comparisons of latent means. Importantly, we do not interpret country-level differences in mean free will belief. Rather, we use country-aggregated free will belief scores as predictors of country-level outcomes that are not themselves multi-item latent constructs (incarceration rates and a single-item measure of death penalty endorsement). Thus, because we are not comparing country-level latent means of free will belief nor modelling multi-item outcome variables that rely on scalar invariance, the lack of scalar invariance for the free will belief predictor is less likely to seriously distort our country-level associations. Nevertheless, this limitation should be borne in mind when interpreting results at the country level.

**Punitiveness** A one-factor model was also tested for the punitiveness scale across all 44 countries. The configural model showed acceptable fit (CFI = .924, RMSEA = .071, SRMR = .054), indicating a stable factor structure across countries. The metric model showed a modest decline in fit (CFI = .909, RMSEA = .070, SRMR = .080), with ΔCFI = −.0158, ΔRMSEA = −.0012, and ΔSRMR = +.0258. Although the ΔCFI slightly exceeded the conventional threshold, the other indices remained within acceptable bounds, supporting a cautious interpretation of metric invariance. Scalar invariance, however, was clearly not supported (CFI = .644, RMSEA = .126, SRMR = .139), with large declines across all indices (ΔCFI = −.265; ΔRMSEA = +.056; ΔSRMR = +.0585).

As with free will belief, scalar invariance for punitiveness is not required for our analyses, as we do not compare mean levels of punitiveness across countries. Rather, punitiveness is modelled as an individual-level dependent variable. Metric invariance is therefore sufficient to support meaningful interpretation of these relationships in our models.

**Extra Analysis Per Reviewer Request (GDP)**

Following reviewer request, we included GDP per capita (purchasing power parity) as a country-level covariate in all analyses. This measure reflects economic output adjusted for inflation and the relative cost of goods and services, expressed in constant 2017 international dollars (World Bank, 2024). Given the strong positive skew typical of GDP data, values were log-transformed prior to analysis. As demonstrated below, the inclusion of this covariate did not meaningfully change the pattern of results.

**Punitiveness.** A linear mixed-effects model was conducted to examine the association between free will belief (within- and between-country components) and punitiveness, controlling for age, gender, religiosity, GDP PPP per capita, and political orientation (see Table S2). Stronger belief in free will was associated with harsher punishment judgments at both the within- and between-country levels.

**Table S2**

*Linear Mixed Model Examining the Effect of Free Will Belief on Punitiveness*

| ***Predictors*** | ***b*** | | | ***95% CI*** | | ***p*** |
| --- | --- | --- | --- | --- | --- | --- |
| (Intercept) | | 6.55 | 4.88 – 8.21 | | | <.001*** |
| Free will belief (within-countries) | | 0.16 | 0.06 – 0.26 | | | .003** |
| Free will belief (between-countries) | | 0.11 | 0.09 – 0.13 | | | <.001*** |
| Gender (female) | | 0.05 | 0.00 – 0.09 | | | .037* |
| Age | | 0.07 | 0.05 – 0.09 | | | <.001*** |
| Importance of religion | | 0.18 | 0.15 – 0.21 | | | <.001*** |
| Economic conservativism | | -0.00 | -0.04 – 0.03 | | | .819 |
| Social conservativism | | 0.06 | 0.02 – 0.09 | | | .001** |
| GDP PPP per capita (log) | -0.21 | | -0.37 – -0.06 | | | .009** |
| ***Random Effects*** |  | |  | | |  |
| Residual | 1.03 | |  | | |  |
| Country (intercept) | 0.06 | |  | | |  |
| ICC | .05 | |  | | |  |
| Observations | 7970 | | | |  |  |
| Marginal R^2^ / Conditional R^2^ | .134 / .179 | | | |  |  |

*Note:* Gender was coded as male (1) and female (2). Marginal R^2^ refers to fixed effects only and Conditional R^2^ refers to the entire model. Predictors were standardized prior to analysis; coefficients represent the expected change in punitiveness associated with a one standard deviation increase in the predictor. * *p* < .05; ** *p* < .01; *** *p* < .001

**Death Penalty Endorsement.** A linear regression revealed no significant effect of mean free will belief on citizen endorsement of the death penalty, *b* = 0.46, *SE* = 0.86, *p* = .593. Inspection of residual plots and formal diagnostic tests indicated no violations of linearity, normality, homoscedasticity, or independence assumptions.
